# Benchmarking Georgia's palliative care system, using WHO's new actionable indicators: alignment with the European Association for Palliative Care (EAPC) 2025 atlas

**DOI:** 10.3389/frhs.2026.1837167

**Published:** 2026-07-10

**Authors:** Tamriko Bulia, Tina Beruchashvili, Ioseb Abesadze, Amiran Gamkrelidze, Pati Dzotsenidze, Koustuv Dalal

**Affiliations:** 1 School of Health Science, University of Georgia, Tbilisi, Georgia; 2 Department of Oncology, Tbilisi State Medical University, Tbilisi, Georgia; 3 Faculty of Medicine, Georgian National University, Tbilisi, Georgia; 4 Division of Public Health Science, Department of Health Sciences, Mid Sweden University, Sundsvall, Sweden

**Keywords:** EAPC, opioids, palliative care services, state programs and policies, WHO

## Abstract

**Introduction:**

Palliative care is an essential health service for patients with chronic and life-threatening conditions. Although palliative care has been formally recognized in Georgia's legislation and state programs since the mid-2000s, evidence regarding the development, accessibility, and integration of services remains limited. The WHO framework, *Assessing the Development of Palliative Care Worldwide: A Set of Actionable Indicators*, and its application in the EAPC Atlas of Palliative Care in the European Region 2025 provide an opportunity for a comprehensive assessment of the national palliative care system. This study evaluated the development of palliative care in Georgia using WHO actionable indicators and compared the findings with the EAPC 2025 Atlas to identify system-level gaps and priorities for improvement.

**Methods:**

This study employed a descriptive health systems assessment and policy analysis of palliative care development in Georgia. The evaluation was guided by the World Health Organization's 2021 framework, *Assessing the Development of Palliative Care Worldwide: A Set of Actionable Indicators*. Fourteen indicators across six domains were operationalized using national administrative data, policy documents, institutional reports, and published literature from 2021 to 2024. Quantitative and qualitative findings were triangulated and benchmarked against the European Association for Palliative Care (EAPC) Atlas of Palliative Care in the European Region 2025 to assess service availability, accessibility, geographical distribution, coverage, and system capacity.

**Results:**

While palliative care is legally recognized and partially embedded in national health policy, implementation remains limited and uneven. Service provision is highly centralized in Tbilisi, with restricted outpatient and home-based services in regions. In 2024, only 16.7% of the estimated national palliative care need was met. Opioid consumption remains in the very low range, reflecting restrictive regulations, limited medicine availability, and insufficient prescriber training. Similarly, the EAPC 2025 Atlas shows low performance across key WHO indicators, particularly in governance, monitoring mechanisms, service integration, research, and education.

**Conclusion:**

Despite early legislative advances, Georgia's palliative care system remains fragmented and inadequately integrated into primary health care. Strengthening governance, financing, education, and monitoring in line with WHO and EAPC benchmarks is essential to achieve equitable, sustainable, and comprehensive palliative care coverage.

## Introduction

Palliative care is a critical component of health systems that aims to improve quality of life by preventing and alleviating physical, psychosocial, and spiritual suffering among patients with chronic and life-threatening conditions (1, 2). Globally, the need for palliative care continues to grow, with recent estimates indicating that more than 70 million people experience serious health-related suffering annually (3). However, only a small fraction receive adequate services (4). The demand for palliative care is expected to rise due to aging populations and the growing burden of non-communicable diseases, emphasizing its importance as a global public health priority (1). A WHO study encompassing 201 countries classified palliative care development into four levels—Emerging, Progressing, Established, and Advanced—revealing that only 14% of countries had reached the Advanced stage (4). This gap underscores profound inequities in access and quality across regions, particularly in low- and middle-income countries (LMICs).

In Georgia, as in most post-Soviet countries, palliative care began to emerge in the early 2000s. The centralized health care system, focused solely on treatment and neglecting holistic approaches, has significantly hindered the field's development, leaving thousands of patients without adequate care (5).

In 2001, Georgian medical NGOs organized a regional conference, inviting palliative care leaders from Hungary, which served as a foundation for international networking and local advocacy. The Open Society-Georgian Foundation (OSGF) played a key role in supporting early development efforts (6).

Initial steps included the opening of the first specialized clinic for incurable cancer patients in 2003. Since 2006, the state program “Palliative Care of Incurable Patients” has provided services to patients with chronic, incurable conditions. Legislative milestones were achieved in 2007, with amendments to three key laws: “Law on Health Care,” “Law on Patients’ Rights,” and “Law on Medical Practice”, which formally defined palliative care as an essential component of health care and established state obligations regarding opioid availability (7). Despite these achievements over the past 25 years, palliative care in Georgia remains fragmented, inconsistent, and predominantly biomedical, with limited integration of holistic, patient-centered approaches. Key barriers include gaps in implementation, limited service integration, and prevailing attitudes toward the medical use of opioids.

Evidence supporting this assessment can be drawn from two key indicators of palliative care accessibility: 1. the number of services provided, and 2. the consumption of opioids for pain relief, the most common symptom among terminally ill patients (8). Since 2004, studies assessing palliative care development in Georgia have primarily relied on these indicators, with a general assessment conducted in 2021 (7). In 2021, the WHO introduced a new conceptual framework for palliative care assessment—Assessing the Development of Palliative Care Worldwide: A Set of Actionable Indicators (9). This framework was subsequently applied by the European Association for Palliative Care (EAPC) in its Atlas of Palliative Care in the European Region 2025, which included Georgia (10). These developments enabled a more comprehensive assessment of the national palliative care system and the identification of existing gaps.

## Aim

This study aimed to benchmark Georgia's palliative care system using WHO's new actionable indicators and to evaluate its alignment with the EAPC 2025 Atlas. By doing so, the study provides a structured and comparative overview of service availability, accessibility, geographical distribution, coverage, and system capacity, while identifying key system-level gaps and priorities for future policy and service development.

## Methods

### Study design

This study was designed as a descriptive health systems assessment and policy analysis of palliative care development in Georgia. The evaluation combined a secondary data review of administrative, policy, and published documents with comparative benchmarking against international indicators. The study aimed to assess the organization, accessibility, capacity, and development of palliative care services and to identify system-level gaps and opportunities for improvement.

### Analytical framework

The evaluation was guided by the World Health Organization (WHO) conceptual framework, “Assessing the Development of Palliative Care Worldwide: A Set of Actionable Indicators” (9). A three-step approach was applied. First, national palliative care indicators were mapped against the six domains of the WHO framework. Second, quantitative evidence was synthesized to assess Georgia's palliative care system's performance across each domain. Third, findings were benchmarked against indicators reported in the European Association for Palliative Care (EAPC) Atlas of Palliative Care in the European Region 2025 (10) to identify strengths, gaps, and priority areas for policy development and service improvement.

### Framework and indicators

The WHO framework comprises 14 indicators organized into six thematic domains: health policies, integrated palliative care services, use of essential medicines, education and training, empowerment of people and communities, and research (9).

Each indicator was operationalized using measurable variables (e.g., number of services, opioid consumption, training programs) and aligned with available national data. The health policy domain was assessed through legislation, national programs, strategic documents, and financing mechanisms. Service provision was evaluated through the number, type, capacity, and geographical distribution of palliative care services. Access to essential medicines was assessed through opioid availability, consumption, and regulatory provisions. Education and training indicators were evaluated through formal educational programs, professional training opportunities, and workforce development initiatives. The domains of community empowerment and research were assessed through a review of relevant institutional reports and published literature on palliative care development in Georgia. Indicators were benchmarked against the EAPC Atlas (10), which applies a comparable WHO-based assessment framework.

### Data sources and validation

Data were collected for the period 2021–2024 from multiple secondary sources, including governmental documentation, administrative datasets, institutional reports, and published literature. The analysis relied exclusively on secondary administrative, policy, and published data sources.

Policy documents, such as laws and regulations, were obtained from the Ministry of Internally Displaced People from Occupied Territories, Labor, Health, and Social Affairs of Georgia (11) (hereafter referred to as the Ministry of Health), the Legislative Herald of Georgia (12), and the Committee on Health and Social Affairs of the Parliament of Georgia (13); Statistical data about morbidity and mortality were obtained from the National Statistics Office of Georgia (14); Data about palliative care service provision and geographical distribution were obtained from the National Health Agency (15) and the Ministry of Health (11). Data about opioid consumption were obtained from the International Narcotics Control Board (INCB) reports (16).

Additional information was obtained from national and international publications, including reports from the World Health Organization (WHO), the International Narcotics Control Board (INCB), the Worldwide Hospice Palliative Care Alliance (WPCA), and peer-reviewed studies relevant to palliative care development.

Data reliability was enhanced through triangulation across multiple independent sources. Quantitative indicators were cross-checked, where possible, using governmental databases, institutional reports, and international datasets to improve consistency and validity.

### Data analysis

To estimate palliative care need, we applied the Worldwide Palliative Care Alliance (WPCA) methodology, which assumes that 37.4% of all deaths are associated with conditions requiring palliative care (8). For each study year, the estimated number of people requiring palliative care was calculated by multiplying Georgia's total registered deaths by 0.374. Annual mortality data were obtained from the National Statistics Office of Georgia (Geostat) (14). The percentage of need met was calculated by dividing the number of patients who received palliative care services by the estimated number of people requiring palliative care and multiplying the result by 100.

To estimate opioid requirements for terminally ill patients, we used the methodology described in the Guide on Estimating Requirements for Substances under International Control, developed by the INCB in collaboration with the WHO (16).

Quantitative indicators included the number of palliative care services, available beds, annual service capacity, service utilization, workforce indicators, and opioid consumption. Data were grouped by service type, geographical location, and calendar year to identify trends and changes over time. Descriptive statistical analyses were performed using Microsoft Excel 2023 (Microsoft Corporation, USA).

Qualitative observations, including service fragmentation, legislative gaps, financing constraints, workforce shortages, and regional inequalities, were synthesized from policy documents and published reports and interpreted alongside quantitative findings.

Findings were subsequently compared with corresponding indicators reported in the EAPC Atlas of Palliative Care in the European Region 2025 (10). This benchmarking process enabled the identification of strengths, gaps, and priority areas for the further development of palliative care services in Georgia.

Quantitative and qualitative findings were triangulated to provide a comprehensive assessment of the development, accessibility, and capacity of palliative care services in Georgia.

#### Results

### Health policies (indicators 3,4)

The development of palliative care policy and legal frameworks began in 2004. With the assistance of WHO palliative care expert Jan Stjernsward, the national action plan was developed. In 2005, the first palliative care program was established (17), followed by supporting changes at a legislative level, introducing palliative care as an integral part of medicine, and reflecting it in the following documents:

1. Law of Georgia on Medical Practice (18).
2. Law of Georgia on Health Care (19).
3. Law of Georgia on Patient Rights (20).
4. Law of Georgia on Narcotic Drugs, Psychotropic Substances and Precursors, and Narcological Assistance (21).

The most remarkable amendments made to legislative documents are the palliative care definitions in two Laws of Georgia (19, 20). The definition emphasizes the field's active, multi-profile approach and holistic nature. The definition was amended in 2019, changing the term “incurable” to a phrase: “With chronic or/and life-threatening disease” (20). With all these amendments, palliative care was recognized as a continuous, coordinated, and comprehensive medical service for the population. Law of Georgia on Health Care defines primary health care as “continuous, comprehensive and coordinated medical services, primarily based on a system of family medicine, available for each member of society”, which also implies palliative care, and ensuring accessibility to essential medicines (21). In 2007, the government undertook the obligation to ensure accessibility of relevant doses and forms of opioids for patients.

The “Law of Georgia on State Budget” allocates funding for palliative care services outlined in the state program (inpatient and outpatient palliative care; “provision of narcotic painkillers”). The state program “Palliative Care for Incurable Patients” has been operating in Georgia since 2006 (22). The program fully covers the cost of opioid medications, reimburses 80%–90% of inpatient palliative care costs, and provides home care for patients with a life expectancy of 3–6 months or less. The program's reimbursement rates are 11 GEL (approximately USD 4) per outpatient visit and 75 GEL (approximately USD 27.5) per inpatient day. Funding continues to rely on reimbursement rates set in 2004, which have not been adjusted for inflation. These limitations highlight systemic underinvestment and weak implementation of palliative care policy.

The Ministry of Health Care of Georgia adopted the first instruction for palliative care provision (23) in 2007, approved the guidelines “Chronic pain management for palliative patients” (24) in 2012, and, in 2019, “Palliative Care for Patients with Chronic Incurable Diseases” (25).

Despite these advances, implementation has been inconsistent. The 2012 National Program for Palliative Care Development (2011–2015) was adopted but not executed (26). More recent action plans (2021–2025) developed by NGOs remain unendorsed by the Ministry of Health.

Policy implication: Legislative recognition has not translated into effective implementation. Without updated financing mechanisms and stronger governance, Georgia's palliative care system risks stagnation.

The medical use of narcotic drugs is strictly regulated by a document, signed and adopted in 2000 under the signatures of two Ministers (27),. According to the regulatory document, prescription of opioids to outpatients for pain relief is allowed only for terminally ill patients. The days for dispensing prescriptions and getting opioids are fixed and mostly possible once or twice a week. According to the regulations, opioids are dispensed from the “pharmacies” located at police departments, and pharmacists dispense a 7-day supply (16). Several amendments were made in 2008 (order 157/n) (23). According to it, the responsibility for prescribing and defining the dosage and forms of opioids was transferred to a single doctor instead of a commission. In addition, to improve geographical accessibility, in 2010, village primary care doctors were authorized to dispense prescriptions on a single-handed basis.

### Access to medicines (indicators 8,9,10)

The majority of the medicines on the list of palliative care essential medicines are available to patients, except those under international control.

Opioid pain-relief medicines are irreplaceable for the management of average and severe pain, and without them, it is often practically impossible to relieve pain and suffering. As stated above, the norms of opioid regulations are quite strict and hinder their prescription on time and when needed. When assessing a patient's condition/prognosis (should be terminal), to avoid a mistake, these regulations require doctors not to prescribe opioids or prescribe them too late.

The second problem relates to the range of medicines available. In Georgia, morphine is the only opioid accessible for outpatient patients with incurable conditions and is currently provided in both injectable and oral (immediate- and prolonged-release tablet) forms. For many decades, only injectable morphine was available in the country, which has shaped prevailing prescribing practices. As a result, many physicians continue to rely on injectable formulations, reflecting limited experience and confidence in pain management using oral morphine, as well as insufficient awareness of its current availability, leading to under-prescription and a lack of formal requests for supply. Taken together, these factors indicate persistently poor opioid consumption in Georgia, placing the country in the very low consumption category according to the INCB, with recent estimates averaging approximately 66 S-DDD (28).

Policy implication: Regulatory barriers and prescribing culture perpetuate undertreatment of pain. Reforming opioid policies and expanding physician training are critical to improving symptom management.

### Service provision (indicators 4, 13, 14)

In Georgia, palliative care includes both outpatient and inpatient services. A total of 19 inpatient palliative care facilities operate nationwide, of which 11 are located in the regions and 8 in Tbilisi. Outpatient units are available in only three regions, leaving the remaining eight regions without outpatient palliative care services (Table 1).

**Table 1:** Distribution and population-based availability of palliative care services and inpatient PC beds in Georgia.

Region/area	Tbilisi	Other regions	Total Georgia
Inpatient PC services (11, 15)	8 (42.1%)	11 (57.9%)	19 (100%)
Inpatient PC beds (11, 15)	247 (74.6%)	84 (25.4%)	331 (100%)
Beds per service^a^	30.9	7.6	17.4
Population (29)	1,331,485	2,598,096	3,929,581
Population per bed^a^	5,391	30,930	11,872
Outpatient PC Services (11, 15)	2 (50.0%)	2 (50.0%)	4 (100%)
Population per outpatient service^a^	665,743	1,299,048	982,395

PC, palliative care.

a Calculated by the authors based on data from references (11, 15, 29).

Sources: References (11, 15, 29).

Although 57.9% of inpatient palliative care facilities are located outside Tbilisi, almost three-quarters of all inpatient palliative care beds (74.6%) are concentrated in the capital. This indicates that the distribution of services is uneven not only in terms of facility numbers but also in terms of service capacity. Outpatient palliative care services are particularly limited, with only four units nationwide, suggesting significant regional disparities in access to care.

According to the 2024 population census, 33.9% of Georgia's population resided in Tbilisi and 66.1% in the remaining regions. Despite this population distribution, 74.6% of all inpatient palliative care beds were concentrated in the capital. The population-per-bed ratio was 5,391 persons per bed in Tbilisi compared with 30,930 persons per bed in other regions, indicating substantial geographical inequalities in inpatient palliative care capacity. Similar disparities were observed for outpatient services, with one palliative care outpatient service serving 665,743 people in Tbilisi compared with 1,299,048 in the remaining regions.

There are six state-funded pediatric palliative care units and one non-state-funded children’s hospice, Firefly World, which provides high-quality, holistic care for inpatient and ambulatory pediatric patients.

The dynamics of service coverage between 2021 and 2024 indicate that service utilization was substantially higher in the capital than in the regions. According to the National Statistics Office of Georgia, the country's population was estimated at 3.914 million on January 1, 2024, of whom 1.330 million resided in Tbilisi and 2.584 million in the remaining regions of the country (29). Despite this population distribution, 83.1% of all inpatient palliative care services provided in 2024 were delivered in Tbilisi (Table 2). For outpatient services, a declining trend was observed in 2024 compared with previous years, primarily due to reduced service provision in the regions.

**Table 2:** Inpatient admissions, outpatient visits, and population-standardized utilization rates by region and year in Georgia.

Year	2021		2022		2023		2024	
Type of service	IA	OV	IA	OV	IA	OV	IA	OV
Tbilisi								
Number of admissions/visits (11, 15)	1,307	309	1,463	296	1,688	332	1,992	305
Population-standardized utilization rate per 100,000^a^	98.2	23.2	109.9	22.2	126.8	24.9	149.6	22.9
Other regions								
Number of admissions/visits (11, 15)	209	150	437	169	303	135	405	55
Population-standardized utilization rate per 100,000^a^	8.0	5.8	16.8	6.5	11.7	5.2	15.6	2.1
Georgia								
Number of admissions/visits (11, 15)	1,516	459	1,900	465	1,991	467	2,397	360
Population-standardized utilization rate per 100,000^a^	38.6	11.7	48.4	11.8	50.7	11.9	61.0	9.2

IA, inpatient admissions; OV, outpatient visits.

a Population-standardized utilization rates per 100,000 population were calculated by the authors based on data from references (11, 15, 29).

Sources: References (11, 15); population data from reference (29).

Population-standardized utilization rates demonstrated substantial regional inequalities in palliative care service use. In 2024, the inpatient admission rate was 149.6 per 100,000 population in Tbilisi compared with 15.6 per 100,000 in the remaining regions. Similarly, outpatient visit rates were 22.9 and 2.1 per 100,000 population in the capital and elsewhere, respectively, indicating markedly higher utilization of palliative care services in the capital.

Between 2021 and 2024, the number of inpatient palliative care services increased steadily, particularly in Tbilisi. In contrast, outpatient service utilization remained relatively stable until 2023 but declined in 2024, primarily due to reduced service provision across regions. As a result, regional differences in service utilization became more pronounced during the study period.

A comparison of the need for palliative care and actual coverage (Table 3) showed that the number of patients receiving palliative care has been increasing annually, although the overall coverage rate relative to estimated need remains low.

**Table 3:** Estimated palliative care need and actual service coverage in Georgia (2021–2024).

Year	Total deaths (14)	Estimated need for PC (37.4%) (8)	Patients who received PC (11, 15)	Need met (%)^a^
2021	59,906	22,405	1,975	8.9%
2022	49,118	18,370	2,365	12.9%
2023	42,756	15,990	2,458	15.4%
2024	43,971	16,445	2,757	16.7%

PC, palliative care; WPCA, Worldwide Palliative Care Alliance.

a Need met (%) was calculated by the authors as: patients who received palliative care ÷ estimated need   ×   100.

Estimated need = total deaths   ×   0.374 (WPCA methodology).

Sources: Mortality data from reference (14); palliative care need estimates based on WPCA methodology (8); palliative care service utilization data from references (11, 15).

Although the number of patients receiving palliative care increased from 1,975 in 2021 to 2,757 in 2024, service coverage remained below the estimated level of need. In 2024, 16.7% of individuals estimated to require palliative care received services through the formal health care system.

### Education (indicators 11,12)

The concept of palliative care initially reflected the biopsychosocial and spiritual aspects; the field's development was followed by the creation of educational resources in Georgian. Since 2005, palliative care manuals for doctors and nurses, a national guideline and standards for pain management, and several monographs combining principles of palliative care across various clinical contexts have been published.

By the end of the first decade of Palliative care development in Georgia, the subject “Palliative Care” was established as an elective course at Tbilisi State Medical University and as a mandatory course at the Medical Faculty of Tbilisi State University. Today, it is offered as a mandatory course at 6 universities and as an elective at 11 universities.

Since 2014, Tbilisi State University has provided the National Palliative Care Association with a 4-month sub-specialty course in palliative care that combines theoretical instruction with supervised clinical practice in inpatient palliative care settings. At the same time, Tbilisi State Medical University, under the Continuous Medical Education Institute, provides short-term programs: “general principles of palliative care,” “Chronic pain management, and communication.”

Education has been significantly supported and enhanced by NGOs, especially by the “Cancer Prevention Center”, which, during the 2013–2017 period, delivered training for primary health care doctors and nurses nationwide, with the support of Caritas Czech Republic.

In total, across 38 medical schools, 17 offer a palliative care course for undergraduate students: 6 as a mandatory course and 11 as an elective. Educational activities in palliative care include subspecialty training programs, continuing medical education courses, and educational initiatives supported by non-governmental organizations. These activities have been implemented alongside the development of palliative care services in Georgia.

### Research (indicator 7)

Research on palliative care in Georgia started in 2008, in collaboration with international partners, to evaluate the level of service development, its accessibility, and regulatory mechanisms.

In 2018, the Journal of Pain and Symptom Management published an article presenting a systematic overview of the palliative care development in Georgia (6), followed by several dissertations and related articles around the topic (30, 31). The assessment of services was conducted and published in 2021 (7) and 2023 (32), once again revealing the need for systemic improvement.

Dissertations and Master's theses indicate a growing interest at the academic level. However, only a few clinical trials, particularly in pain management, symptom therapy, and holistic approaches, are currently available (see Appendix N1 for details). In addition, no coordinated national research strategy for systematic data collection and application in policy development exists. Research activity remains concentrated primarily in academic publications and postgraduate research projects.

### Community (indicator 1)

Several public awareness-raising activities were carried out during the palliative care implementation process. Leading organizations have conducted several activities, directed at the enhancement of knowledge and awareness among the medical personnel – workshops, conferences, and masterclasses were conducted with the involvement of international experts. Open-door public days in Hospices, educational lectures, charity marathons and exhibitions, as well as informational campaigns, were organized to support access to medicines and effective management of pain. All these activities were organized by leading palliative care organizations in collaboration with NGOs (particularly OSGF/OSI) and medical institutions.

### Palliative care monitoring indicators - EAPC (indicators for monitoring PC development) comparative analysis

As mentioned above, in our study, the EAPC Palliative Care Atlas served as an additional source for a comprehensive comparative analysis of palliative care services in Georgia (10). Below, the EAPC ratings for each indicator are presented, accompanied by comments where further clarification is warranted.

Comparison of the study findings with the EAPC Atlas demonstrated substantial agreement across most indicators of palliative care development in Georgia. Consistent findings were observed regarding the recognition of palliative medicine as a medical subspecialty, the availability of specialized palliative care services, educational activities, and the absence of formal national coordination mechanisms. The comparison also highlighted differences in the assessment of opioid availability and consumption, research activity, pediatric palliative care services, and regional distribution of services. Overall, both assessments indicate that palliative care development in Georgia remains uneven across different domains, with progress achieved in some areas and persistent gaps in others.

## Discussion

### Key findings

This study demonstrates that while Georgia has made progress in establishing a legislative and policy framework for palliative care, service provision remains fragmented, underfunded, and heavily centralized in Tbilisi. Coverage relative to need is critically low, with only 16.7% of the estimated population requiring palliative care receiving services in 2024. Opioid accessibility is severely restricted, and education and research remain limited. Benchmarking against WHO indicators and the EAPC 2025 Atlas confirms Georgia's classification as “Progressing,” with little systemic improvement over the past decade.

### Health policies and palliative care services

In Georgia, palliative care policy development began in 2004 and advanced after 2007 through incorporation into national legislation. Palliative care was recognized as a continuum and coordinated medical service that should be integrated into primary health care and be accessible to all individuals in need. Subsequent legislative amendments further emphasized the holistic and multidisciplinary nature of care, as well as the importance of early identification and prevention of suffering (27). According to the law, beneficiaries can be “patients with chronic or life-threatening disease,” (20). However, legislative recognition has not translated into effective implementation. The key challenges of the system are its fragmentation and limited access to services. The State Program for Palliative Care does not fully implement a holistic approach and only covers patients’ medical needs. The program strictly defines beneficiaries as terminal-stage incurable patients, poses limitations on early-stage outpatient care, stipulating “any attempt at further treatment is unreasonable” (26). Such an approach undermines continuous and coordinated care and conflicts with the aforementioned legal provisions regarding timely access to services. Treatment for outpatients is capped strictly at six months, which contradicts the Law of Georgia, which defines palliative care as a needs-based, integrated service for patients with chronic or life-threatening conditions, rather than solely as end-of-life care (7). Due to fear of possible penalties, physicians often include patients in the program only when their physical condition has deteriorated severely, leaving little chance for improvement in functional status and, consequently, limited benefit from the program. This tendency is compounded by the fact that many physicians lack adequate knowledge of prognosis and, as is well known, generally overestimate patients’ life expectancy.

The same applies to Indicator 4, which concerns the inclusion of palliative care at the primary care level. The EAPC rates Georgia at level 3, noting that the program aims “to improve quality of life by increasing financial access and providing specific medications.” However, the program's above-mentioned restrictions regarding diagnosis and life expectancy—particularly the limitation that opioids may only be prescribed to terminally ill patients when no disease-oriented treatment options remain—prevent not only early-stage but also timely service provision to outpatients. Furthermore, patients’ and families’ expectations of continued disease-oriented treatment often block access to strong pain medications needed to relieve suffering until the end of life, thereby limiting opportunities to improve quality of life through timely symptom management.

Another major challenge is inadequate financing. Funding of the program continues to rely on outdated reimbursement rates established in 2004, which have not been adjusted to account for substantial inflation, particularly during the past decade (33). The program covers home-based services delivered by primary care staff; however, four facilities currently provide them due to limited funding. Consequently, the number of patients receiving home-based palliative care declined from 900 in 2018 to 360 in 2023 (34). This represents approximately 13% of all palliative care services provided and only about 2% of the estimated national need (43,971 deaths in 2024; estimated palliative care need: 16,445 patients; 37.4%) (Table 3). Inpatient services cover an additional 15% of the estimated need. Similar levels of palliative care coverage have been reported internationally, with the Lancet Commission on Global Access to Palliative Care and Pain Relief reporting coverage estimates of approximately 17% in comparable settings (35).

Current inflation in the country undermines medical personnel's willingness to work in underfunded palliative care services. The field remains unattractive to medical personnel, affecting both quantitative and qualitative parameters and contributing to the unsustainable, sporadic nature of existing services. Hard socio-economic conditions also drive urbanization. The relatively small number of patients in the regions makes poorly funded services even less attractive.

Geographical accessibility, decentralization, and local personnel development (36) are also problematic. Our study showed drastic centralization of services in the capital, Tbilisi, with 75% of inpatient beds, while only 34% of the population lives there (29). Only 7 of Georgia's 11 regions provide inpatient palliative care services. Only 4 outpatient palliative care services are available nationwide: 2 in Tbilisi and 2 in other regions of the country. Such an imbalance hinders the provision of services to patients with chronic, incurable conditions and conflicts with WHO guidance on community-based, primary care–level integration. National policy should ensure equitable distribution and staffing with qualified personnel (Indicators 13 and 14), thereby enabling full coverage and reducing patient and family suffering.

Other gaps in the program hinder the implementation of a holistic palliative care approach. Although the state budget covers primary outpatient and inpatient costs, it allocates funds for only a doctor and a nurse, making it impossible to fully meet patients’ multifaceted needs (33). No funds are provided for social workers or psychologists. The restrictions on diagnosis and prognosis, together with the program's limitation to medical services only, hinder the possibility of advance care planning (Indicator 2, level 1, Table 4).

**Table 4:** WHO palliative care indicators and EAPC 2025 atlas data for Georgia (10).

N	Indicator description (WHO)	EAPC rating/value	EAPC comments	Authors’ comments
1	Existence of groups dedicated to promoting the rights of patients in need of PC, their families, caregivers, and disease survivors	3	Advocacy mostly by individuals	Findings are consistent with the EAPC assessment
2	Existence of a national policy or guideline addressing advanced care planning of medical decisions for the use of life-sustaining treatment or end-of-life care	1	No national policy or guideline on advance care planning.	Findings are consistent with the EAPC assessment
3	Existence of a current national PC plan, program, policy, or strategy with a defined implementation framework, The national plan includes indicators to monitor and evaluate progress, with measurable targets.	2 1	The country currently operates the state program ‘Palliative Care of Incurable Patients’; the latest strategy was adopted in 2012, but has not been further implemented.	Findings are consistent with the EAPC assessment
4	Inclusion of PC in the list of health services provided at the primary care level within the national health system	3	Included in the essential services list as recognized by government decree or law, but not covered under the General Health Law.	Refers to state programs approved annually by government resolutions (see Discussion).
5	Existence of a national coordinating authority for PC	1	No evidence of a formal coordinating authority for PC	One person in the Ministry of Health's health department oversees medicines procurement for the state program
6	Existence of congresses or scientific meetings at the national level specifically related to PC	2	No evidence found; only outdated congresses	Findings are consistent with the EAPC assessment
7	PC research in the country, measured by peer-reviewed articles, the number of articles published on the topic, or the inclusion of PC topics in national research calls.	1	No evidence	Findings are consistent with the EAPC assessment
8	Reported annual opioid consumption (S-DDD), excluding methadone.	1,176 S-DDD opioid consumption, for statistical purposes in 2020–2022	No evidence	In Georgia, only morphine is used for pain relief (60–66 S-DDD, INCB; see Discussion)
9	Availability of essential medicines for pain and PC at all levels of care	3 (urban) 2 (rural areas)	No evidence	Most of the essential PC medicines, except opioids, are more or less available at the primary care level.
10	General availability of immediate-release oral morphine (liquid or tablet) at the primary level.	1	No evidence	Oral morphine is only available in urban areas.
11	Education and Training: Proportion of medical and nursing schools including PC formal education in undergraduate curricula	6/38 medical schools mandatory; 11 elective	No information on PC education for nurses; two full professors in palliative medicine	Findings are consistent with the EAPC assessment
12	Existence of an official specialization process in palliative medicine for physicians, recognized by the competent authority in the country	4	PC recognized as a subspecialty	Findings are consistent with the EAPC assessment
13	Integrated PC services and specialized palliative care programs	2	23 PC services: 19 inpatient, 4 outpatients, with 14 palliative care teams.	Uneven distribution; concentrated in Tbilisi (Table 1)
14	Number of specialized PC programs for the pediatric population in the country	2	6 state-funded pediatric PC teams	Children's hospice ‘Firefly World’ – high-quality, holistic care; not state-funded

EAPC, European Association for Palliative Care; PC, palliative care; S-DDD, Defined Daily Dose for statistical purposes; WHO, World Health Organization.

Source: EAPC Atlas of Palliative Care in the European Region 2025 (10). Authors’ comments are based on the present analysis and national data sources.

The same is evidenced by the study, conducted in Georgia in 2021, showcasing that among 19 palliative care providing facilities, only 26% (1/4) of them were providing a multidisciplinary approach - only 1 of 7 outpatient facilities by that time had a social worker, and 3 of them formally had a psychologist (7).

The National Program for Palliative Care Development (2011–2015) was adopted but not executed (26). The more recent action plan (2021–2025) developed by NGOs was not endorsed by the Ministry of Health. The unavailability of the national plan and its indicators makes it difficult to monitor and evaluate progress, including measurable targets (indicator 3). The absence of a national coordinating authority (Indicator 5) reflects weak institutional leadership, leaving program oversight largely dependent on isolated administrative functions. Given the low EAPC evaluation (predominantly level 1) for WHO Indicators 2, 3, and 5, which cover the existence of relevant policies, evaluation, and monitoring tools, and national responsibility, these findings suggest that palliative care has not yet been consistently prioritized within health policy implementation in Georgia (Table 4).

Overall, its development has remained at level 2 (EAPC) for nearly two decades (10). Based on the 4-group typology of Hospice and palliative care development elaborated by the International Observatory on End of Life Care of Lancaster University (IOELC), Georgia was assigned to the 3b group, i.e., the country with Generalized Palliative Care Provision (37). The 2025 assessment places it in the same group, indicating no systemic or structural improvements over 14 years.

### Essential medicines and pain management

The accessibility of essential medicines in Georgia can be rated from moderate to good, except for opioids. (Indicators 8, 9, and 10). Pain management practice remains a serious challenge that severely affects access to essential medicines (34). In addition to legal restrictions—such as requirements for diagnosis and prognosis (“terminal stage of incurable diseases” and “no disease-oriented treatment options” remaining), and regulations mandating that opioids be dispensed only once a week with a maximum seven-day supply from police stations—educational gaps, personal attitudes, and systemic biases further hinder access to opioid pain medications. Availability of oral morphine only in urban areas can be explained by the lack of information and “fear” among primary care doctors to prescribe and manage adequate doses of opioids (linked to Indicators 5 and 11). There is no national coordinating authority for palliative care (Indicator 5) to ensure effective information delivery and guarantee that all available medications in the country are accessible nationwide. Not all primary health care doctors involved in palliative care programs are authorized to prescribe morphine—only about 50% have this authority (7). As described in the education section, of the 38 universities in Georgia, only 17 currently offer palliative care training, and in just 35% of these institutions, it is offered as a mandatory course. Moreover, not all curricula address pain management or opioid prescribing practices, further limiting physicians’ competence and confidence in this area. This gap reinforces misconceptions and contributes to reluctance in prescribing opioids when clinically indicated, as demonstrated in other studies, based on primary data (38).

The WHO 2025 Report states that patients should have access to opioids if medically needed (39). EAPC defines opioid consumption for Georgia as 1,176 S-DDD. Although Methadone, used exclusively for substitution therapy, was excluded from the evaluation, the data may not fully reflect reality. In Georgia, only morphine (for outpatients) is used for pain relief, with a total consumption rate of 60–66 S-DDD in the country, placing it in the very low category (1–100 S-DDD) (28). Higher consumption rates in the EAPC Atlas are due to buprenorphine (S-DDD 989) and fentanyl (S-DDD 121), neither of which is used for pain in Georgia. Strict policies, administrative barriers, and a lack of knowledge, awareness, and experience among prescribers mostly influence low opioid use.

### Education and training

The development of palliative care education in Georgia reflects both progress and persistent gaps. Initially, the concept of palliative care was introduced through resources that emphasized its biopsychosocial and spiritual dimensions, including manuals, national guidelines, and monographs published since 2005. These early efforts laid the foundation for integrating palliative care principles into medical training.

By the end of the first decade of palliative care development, courses were introduced at major universities, with Tbilisi State Medical University offering palliative care as an elective and Tbilisi State University establishing it as a mandatory subject. Today, six universities provide mandatory palliative care courses and eleven offer them as electives, representing a notable expansion but still covering fewer than half of Georgia's medical schools. Nevertheless, most medical schools do not include palliative care in their mandatory curricula (29%), and over half omit it in their bachelor programs (55%). This gap indicates that many future physicians in Georgia may enter clinical practice without adequate knowledge of palliative care, potentially affecting service quality, integration, and timely referral to palliative care services. Nursing education remains particularly underdeveloped and is largely absent from formal training programs (Indicator 11).

Specialized training opportunities have also emerged. Since 2014, Tbilisi State University has offered a four-month subspecialty program combining theoretical instruction with supervised clinical practice, preparing physicians for inpatient palliative care services. In parallel, short-term continuing medical education programs on general palliative care principles, chronic pain management, and communication have been delivered through the Continuous Medical Education Institute. Importantly, NGOs such as the Cancer Prevention Center, supported by Caritas Czech Republic, have played a critical role in extending palliative care training to primary care doctors and nurses nationwide. The palliative care sub-specialty, available to Georgian doctors, enhances professional status and ensures high-level training (Indicator 12). However, the limited number of specialists and fragmented education systems impede the delivery of effective, timely care (Indicators 13 and 14). Lack of education is directly linked to poor service provision, especially for pain management (40).

Overall, while Georgia has made important strides in establishing palliative care education, limited coverage, curricular variability, and insufficient emphasis on pain management highlight systemic weaknesses. Strengthening undergraduate and postgraduate training, expanding mandatory palliative care education across all medical schools, and embedding opioid prescribing competencies into curricula are essential steps toward improving service delivery and ensuring equitable access to quality palliative care.

### Research

Research on palliative care in Georgia has evolved slowly but steadily since its initiation in 2008, primarily through collaborations with international partners to evaluate service development, accessibility, and regulatory mechanisms.

The increasing number of dissertations and Master's theses demonstrates expanding engagement at the academic level. However, research activity remains largely confined to academic publications and postgraduate projects, with only a few clinical trials addressing pain management, symptom therapy, or holistic approaches. Importantly, Georgia lacks a coordinated national research strategy to guide systematic data collection and ensure the translation of findings into policy development.

Overall, while research activity has expanded over the past decade, its scope and impact remain limited. Establishing a national research agenda, fostering clinical trials, and embedding research into health policy processes are essential steps toward strengthening evidence-based palliative care and ensuring that services evolve in line with patient needs and international standards.

### Empowerment of people and communities

Low public and professional awareness of palliative care in Georgia remains a critical barrier to the development of palliative care services. This reflects not only a lack of information but also a deep cultural stigma, whereby palliative care is perceived primarily as care associated with death rather than as a holistic approach to improving quality of life. Current policy provisions for palliative care and pain treatment inadvertently reinforce these misconceptions, as restrictive regulations and the absence of advance care planning (Indicator 2) limit patients’ rights and reduce opportunities for comprehensive care.

Advocacy for patient rights is largely individual-led (Indicator 1), with limited institutional or systemic support. This weakens the visibility of palliative care as a legitimate component of health care and perpetuates its marginalization. Although educational initiatives and public awareness campaigns have been implemented in recent years, gaps in understanding the role, availability, and benefits of palliative care persist among patients and families.

Overall, the persistence of stigma, insufficient policy support, and fragmented advocacy hinder the normalization of palliative care within the health care system. Strengthening public education, embedding palliative care into broader health communication strategies, and institutionalizing patient rights advocacy are essential steps toward overcoming misconceptions and ensuring equitable access to palliative care services.

### Comparison with the literature and other post-soviet countries

The findings of this study should also be interpreted within the broader context of palliative care development in post-Soviet countries.

Comparison with Armenia, Moldova, Ukraine, Kazakhstan, and Azerbaijan demonstrates both the progress achieved in Georgia and the persistent challenges shared across the region. According to the EAPC Atlas 2025, Georgia reports the highest rate of specialized palliative care services among these countries (0.61 services per 100,000 population), exceeding Armenia (0.53), Moldova (0.48), Ukraine (0.23), Kazakhstan (0.18), and Azerbaijan (0.01) (10). This suggests comparatively stronger service availability despite Georgia's limited health care resources.

Access to essential medicines represents another area where Georgia performs relatively well. Opioid consumption in Georgia (1,176 S-DDD per million inhabitants per day) substantially exceeds that reported in Armenia (81), Ukraine (132), Kazakhstan (36), and Azerbaijan (30) (10). Although significant barriers to opioid prescribing and utilization remain, the comparatively higher opioid consumption reported in Georgia suggests greater availability of essential medicines for pain management than in several neighboring post-Soviet countries. Nevertheless, the continued underuse of oral morphine and the concentration of prescribing authority among a limited number of physicians suggest that regulatory restrictions, professional attitudes, and insufficient training continue to impede optimal pain control.

At the policy level, Georgia and Armenia demonstrate more advanced development through formal recognition of palliative care as a medical specialty and partial integration into national health systems. In contrast, Kazakhstan, Ukraine, and Azerbaijan continue to show weaker institutional recognition and lower levels of policy development (10). However, regional comparisons also highlight opportunities for improvement. Moldova and Ukraine have achieved more comprehensive integration of palliative care into national health benefit packages, illustrating how policy commitment can facilitate broader access to services and continuity of care. These experiences suggest that strengthening integration within primary health care and expanding palliative care entitlement beyond end-of-life eligibility criteria could further improve access in Georgia.

Workforce development remains a common challenge across the region. Although Georgia has established formal specialist training pathways and academic positions in palliative care, educational opportunities remain fragmented, and mandatory undergraduate training is absent in most medical schools. Similar workforce shortages have been reported throughout post-Soviet countries, limiting service expansion and contributing to geographical disparities in access (10). The concentration of services and specialists in major urban centers reflects a broader regional pattern in which inadequate financing mechanisms and limited professional incentives hinder the development of sustainable palliative care services outside capital cities.

Overall, comparisons with neighboring post-Soviet countries indicate that Georgia has made notable progress in service provision, specialty recognition, and access to essential medicines. However, the experiences of Moldova and Ukraine demonstrate that further advances in service integration, workforce development, and sustainable financing mechanisms are necessary to achieve a more equitable and comprehensive palliative care system. These findings reinforce that the principal barriers to palliative care development in Georgia are no longer legislative recognition alone, but rather the effective implementation, financing, and integration of services across all levels of the health care system. The limited progress observed over the past decade highlights the challenges of translating legislative recognition into effective implementation and sustainable service development.

### System-level barriers

Several system-level barriers continue to limit the development of palliative care in Georgia. These include weak governance and inadequate financing, restricted access to opioid analgesics, regional inequalities in service availability, workforce shortages, educational gaps, and low public awareness. National action plans remain largely unimplemented, while outdated reimbursement rates limit service sustainability. Restrictive opioid regulations continue to impede access to effective pain management, and the concentration of services in Tbilisi contributes to substantial geographical disparities in access. Persistent stigma and the continued association of palliative care with end-of-life care further restrict service utilization and advocacy efforts.

### Policy implications

The findings of this study suggest several priorities for the development of palliative care in Georgia. These include implementing a comprehensive national strategy, integrating palliative care into primary health care, improving access to opioid analgesics, and ensuring a more equitable regional distribution of services. Additional priorities include strengthening multidisciplinary workforce capacity, expanding palliative care education and research, and increasing public awareness to reduce stigma and improve access to services. Collectively, these measures may help address the governance, financing, workforce, and accessibility barriers identified in this study.

### Strengths and limitations

Strengths of this study include the use of internationally recognized WHO and EAPC conceptual frameworks, triangulation of multiple data sources, and benchmarking against international indicators, which enhance the reliability, comparability, and policy relevance of the findings.

Several limitations should be acknowledged. The analysis relied primarily on administrative and documentary data sources, which may be subject to reporting inaccuracies or incomplete records. In addition, the descriptive design does not allow causal inferences regarding the relationship between identified barriers and service outcomes. Patient-level outcomes, including patient satisfaction, caregiver burden, symptom control, and quality-of-life measures, were not evaluated.

Future research should complement health system assessments with patient- and caregiver-oriented outcomes, including access barriers, symptom management quality, patient satisfaction, caregiver burden, and health-related quality of life. Such information would provide a more comprehensive understanding of the effectiveness and quality of palliative care services and help guide patient-centered service development and policy improvements.

Finally, the interpretation of some indicators required contextual judgment, which may limit direct comparability with assessments conducted in other settings.

## Conclusion

Palliative care development in Georgia has advanced through legislative recognition and the establishment of state programs, yet systemic gaps persist in service coverage, financing, opioid accessibility, education, and research. Despite progress in policy frameworks, implementation remains fragmented and disproportionately centralized in Tbilisi, leaving large segments of the population underserved. Benchmarking against WHO indicators and the EAPC 2025 Atlas confirms Georgia's classification as “Progressing,” with limited improvement over the past decade.

Strengthening palliative care requires a comprehensive national strategy that integrates services into primary care, ensures equitable regional distribution, reforms opioid regulations, and expands workforce training. Sustainable financing, investment in research, and community engagement are important components of continued system development and may help reduce stigma and improve patient- and family-centered care. Addressing these priorities could facilitate the development of a more integrated, equitable, and high-quality palliative care system that aligns with international standards and better meets the needs of its population.

## Why was this study carried out?

Palliative care has been legally recognized in Georgia since the mid-2000s, yet no comprehensive benchmarking aligned with the WHO framework and the EAPC 2025 Atlas has been performed. There is a paucity of literature, particularly from LMICs, evaluating palliative care systems and identifying implementation gaps between policy commitments and actual service delivery, accessibility, and integration. This study was conducted to provide a structured and evidence-based assessment of Georgia's palliative care system using the World Health Organization's 2021 actionable indicator framework.

## What was learned from the study?

The study revealed that despite early legislative progress, Georgia's palliative care system remains fragmented, centralized, and insufficiently integrated into primary health care. Benchmarking against the EAPC 2025 Atlas confirmed low performance across key WHO indicators, particularly in governance, monitoring, education, and research. Overall, the findings highlight a substantial gap between policy recognition and practical implementation, underscoring the need for systemic reform and strengthened national coordination. The findings could enrich policymakers in Georgia and other LMICs.

## Data Availability

The original contributions presented in the study are included in the article/Supplementary Material, further inquiries can be directed to the corresponding authors.
