# Supplementary material for: Benchmarking Georgia's palliative care system, using WHO's new actionable indicators: alignment with the European Association for Palliative Care (EAPC) 2025 atlas
Source: Front Health Serv. 2026 Jul 10;6:1837167. doi: 10.3389/frhs.2026.1837167 (PMC13396248; doi:10.3389/frhs.2026.1837167)
Supplement: Supplementary file 1 [file Table1.docx]

Appendix N1. Selected Research on Palliative Care and Health Services in Georgia

| **Study Type** | **Topic** | **Author** | **Year** | **Institution** | **Focus** |
| --- | --- | --- | --- | --- | --- |
| Master’s Thesis | Assessment of Medical Institution Quality and Provision | Rusudan Pataridze, Giorgi Firt’qalaishvili | 2014 | Ilia State University | Quality assessment (Health Policy & Management) |
| PhD Dissertation | The necessity to consider end-of-life perceptions and attitudes when developing a palliative care model for the elderly | Mariam Velijanashvili | 2016 | Ivane Javakhishvili Tbilisi State University | Life-end perceptions and care model development in the elderly |
| PhD Dissertation | End-of-life perceptions and attitudes under conditions of limited life expectancy: A comparative analysis of cancer patients and non-patient groups | Nana Chikhladze | 2017 | Ivane Javakhishvili Tbilisi State University | Perceptions and Attitudes at End of Life in Conditions of Understanding Limited Life Expectancy: Comparative Analysis of Groups of Cancer Patients and Patients without Cancer |
| PhD Dissertation | Assessment of existing barriers in chronic pain management and their impact on the quality of medical care | Pati Dzotsenidze | 2018 | Ivane Javakhishvili Tbilisi State University | Barriers in chronic pain management |
| PhD Dissertation | Analysis of the needs of oncological patients, criteria for assessing palliative care status, and the medical services provided in Georgia | Tamar Lekashvili | 2019 | Ivane Javakhishvili Tbilisi State University | Needs, palliative care status & service analysis |
| PhD Dissertation | Organizational barriers to pain management in the cancer care process | Natia Shavdia | 2022 | University of Georgia | Barriers in chronic pain management |
| Master’s Thesis | Pain management services in Georgia | Shorena Gabedava | 2025 | Ivane Javakhishvili Tbilisi State University | Barriers in chronic pain management |

Full texts available upon request or via institutional repositories.
